# Supplementary figures and images for: Resistance to lethal ectromelia virus infection requires Type I interferon receptor in natural killer cells and monocytes but not in adaptive immune or parenchymal cells
Source: PLoS Pathog. 2021 May 20;17(5):e1009593. doi: 10.1371/journal.ppat.1009593 (PMC8172060; doi:10.1371/journal.ppat.1009593)

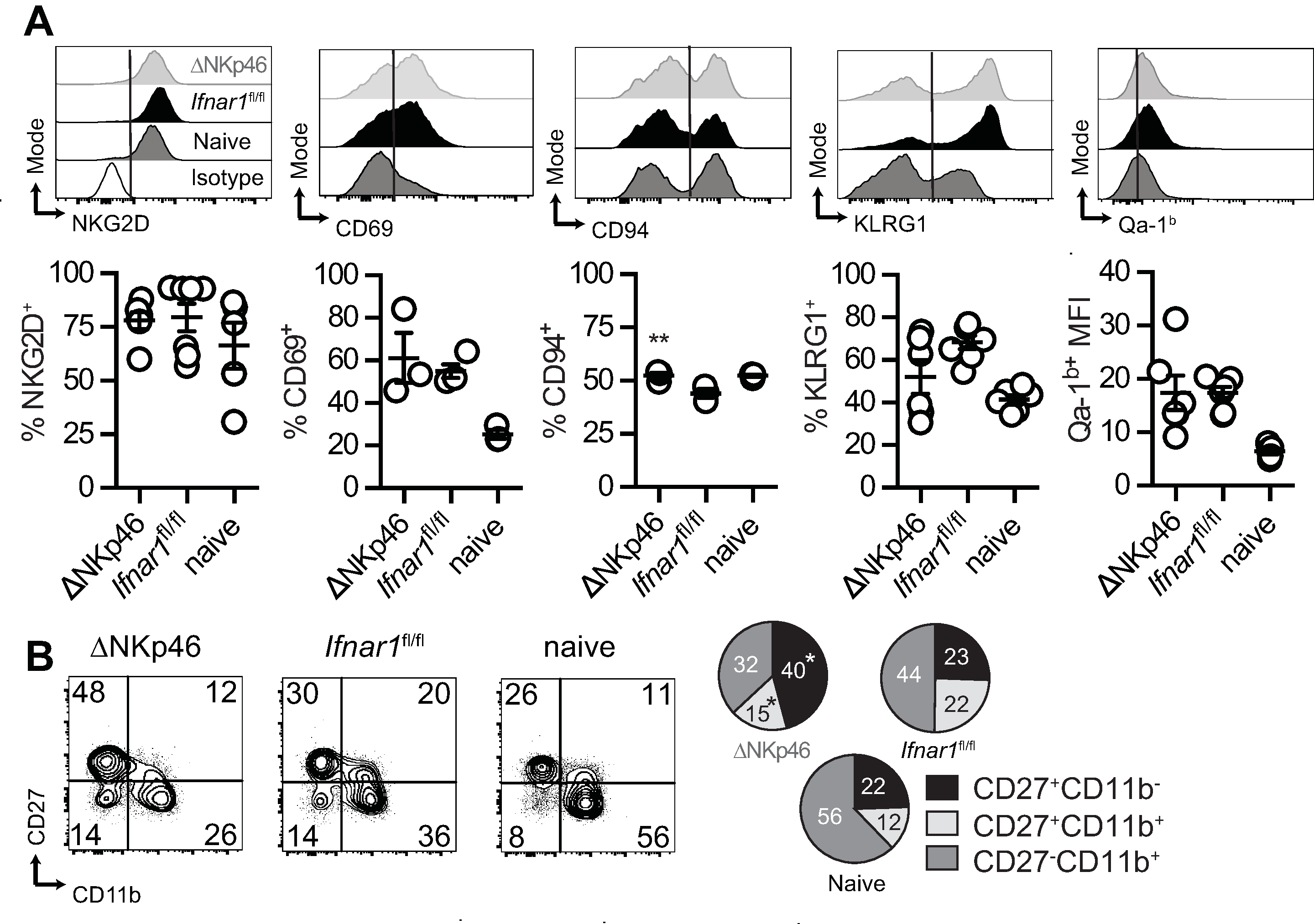

Supplement: S1 Fig — Deletion of IFNAR1 in NK cells results in increased frequency of immature NK cells after ECTV. (A-B) The indicated mice were infected with 3000 pfu of ECTV-GFP in the footpad and spleens were harvested at 6 dpi. (A) Concatenated histograms and graphs showing proportions of KLRG1, NKG2D, CD69 and CD94 expression in gated NK cells (NK1.1+ TCRβ-) and MFI of expression of Qa-1b+ NK cells. (B) Concatenated flow cytometry plots and pie charts showing average proportions of NK cells subpopulations based on CD27 and CD11b expression. Data are represented as mean from two pooled independent experiments (N = 6 or 7 for each group, ANOVA with Tukey correction compared to Ifnar1fl/fl infected mice). (TIF) [file ppat.1009593.s001.tif]

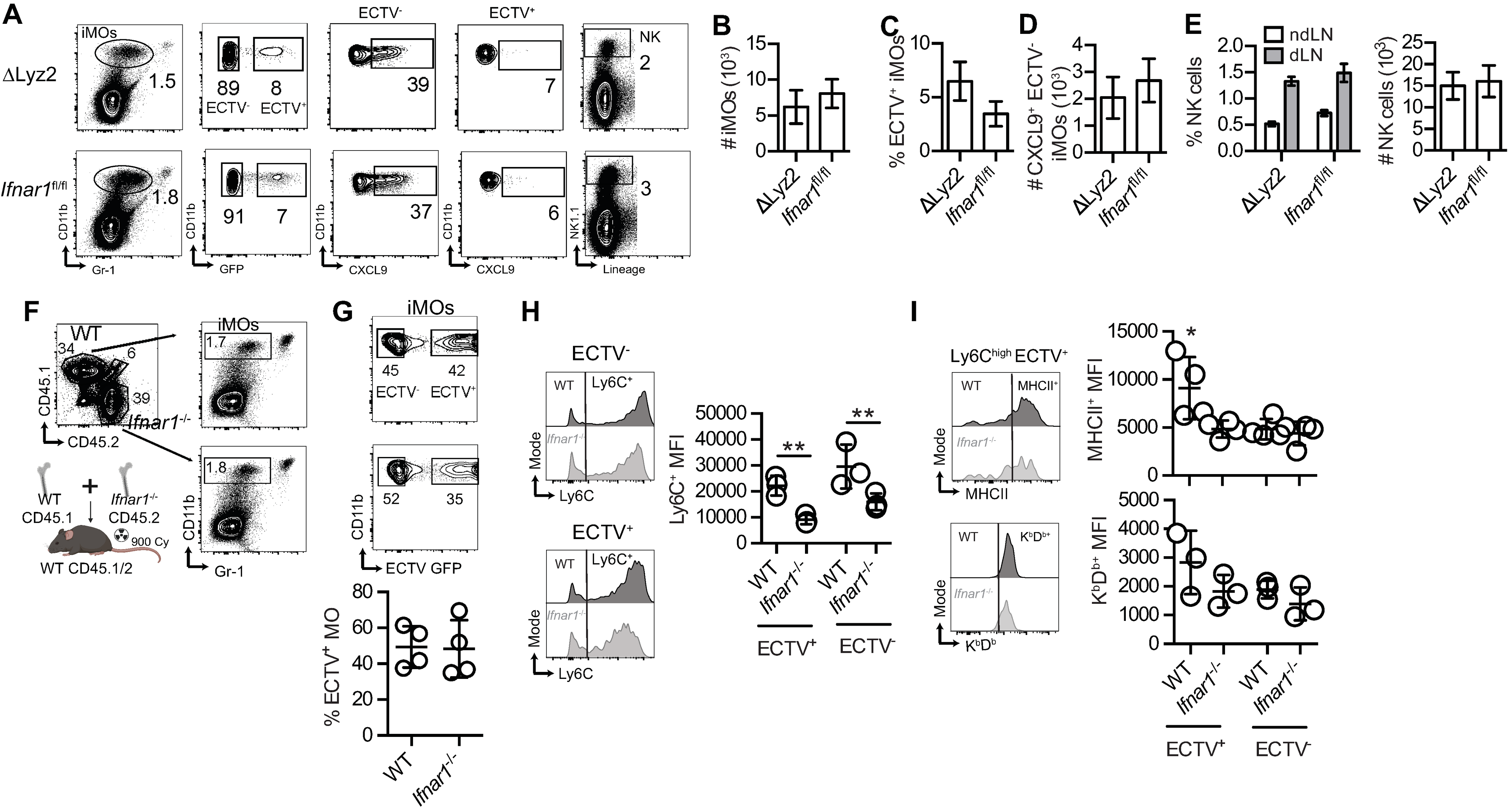

Supplement: S2 Fig — iMOs require intrinsic IFNAR for efficient Ly6C and MHC-II expression but not to migrate to dLNs, produce CXCL9, or resist infection. (A-D) Indicated mice were infected with 3000 pfu of ECTV-GFP in the footpad, and at 2 dpi, the popliteal dLN and collateral non-draining lymph node (ndLN) were harvested. (A) Concatenated flow cytometry plots showing proportions of iMOs (CD8-CD4-NK1.1-CD11b+Gr-1+), proportions of infected (ECTV+), and uninfected (ECTV-) iMOs determined by GFP expression, proportions of CXCL9 expression within ECTV+ and ECTV- iMOs populations, and proportions of NK cells (CD8-CD4-NK1.1+) observed in the dLN. (B) Numbers of iMOs in dLN. (C) Frequency of infected iMOs expressing GFP in dLN. (D) Numbers of uninfected iMOs expressing CXCL9. (E) Frequency and numbers of NK cells in dLN. Data are represented as mean ± SEM from two pooled independent experiments (N = 8–9 for each Cre+ mice and N = 15 for Ifnar1fl/fl mice, ANOVA with Tukey correction compared to Ifnar1fl/fl mice). (F-I) Mixed BMCs B6.CD45.1 + Ifnar1-/-→ F1 [B6.CD45.1 x B6.CD45.2] were infected with 3000 pfu of ECTV-GFP in the footpad and the popliteal dLNs were harvested at 3 dpi. (F) Flow cytometry histograms showing iMOs proportions within WT (B6.CD45.1) and Ifnar1-/- (CD45.2) populations and histograms depicting levels of Ly6C expression in each of these iMOs populations. (G) Flow cytometry plots showing ECTV- and ECTV+ iMOs as determined by GFP expression in WT and Ifnar1-/- iMOs and graph showing the percentages of ECTV+ iMOs. (H) Flow cytometry histograms showing Ly6C expression in infected and uninfected iMOs and mean fluorescence intensity of Ly6C+ in ECTV- and ECTV+ iMOs populations. (I) Flow cytometry histograms showing levels of KbDb MHC-I and MHC-II expression in Ly6C+ ECTV+ iMOs and mean fluorescence intensity of these molecules in ECTV+ and ECTV- Ly6C+ iMOs. Data are represented as mean ± SEM from three pooled independent experiments in which dLNs from individual BMCs (N = 8–10 m [file ppat.1009593.s002.tif]
