# Supplementary material for: Resistance to lethal ectromelia virus infection requires Type I interferon receptor in natural killer cells and monocytes but not in adaptive immune or parenchymal cells
Source: PLoS Pathog. 2021 May 20;17(5):e1009593. doi: 10.1371/journal.ppat.1009593 (PMC8172060; doi:10.1371/journal.ppat.1009593)
Supplement: Attachment — Submitted filename: Melo-Silva PP revised Response 03-30-2021.docx [file ppat.1009593.s003.docx]

# Point-by-Point response to the reviewers’ comments

We thank the reviewers for their insightful observations, which have all been answered below. Reviewers’ comments are in black and our answers are in blue. Within the manuscript, edits in response to the reviewers’ comments are highlighted in yellow.

Reviewer Comments (if any, and for reference):

Reviewer's Responses to Questions

**Part I – Summary**

Please use this section to discuss strengths/weaknesses of study, novelty/significance, general execution and scholarship.

Reviewer #1: This manuscript adresses the requirement of IFN signaling in different immune cell types for resistance to lethal ectromelia virus infection. Whilst the importance of type I IFN in mousepox resistance is well-established, whether all or specialised cellular compartments need IFN-I signalling is unclear, so the biological question underpinning the research is relevant. The findings reported are very interesting and will help us understand how the host immune response is executed and compartimentalised during viral infection, an area to which the senior author of the manuscript has substantially contributed. I have a number of questions/clarifications, two of which (in the major issue section) should be addressed in order to strenghten the study.

Reviewer #2: Melo-Silva et al. revealed by experiments with bone marrow chimeric mice that IFNAR signaling of hematopoitic cells is needed to control mousepox infection. Despite mousepox infection induces hepatitis, IFNAR signaling of hepatocytes did not crucially contribute to protection. Furthermore, IFNAR signaling of T and B cells was not needed for protective T cell responses, whereas intrinsic IFNAR signaling of NK cells as well as of inflammatory monocytes was vital for optimal resistance.
This study shows important new and surprising data of how type I IFN confers resistance to mousepox infection. In this study experiments with classical bone marrow chimeric mice and more advanced conditional knock out mice were combined to elegantly work out the specific role of NK cells and inflammatory monocytes. The experiments were thoughtfully planned carefully carried out. It is appreciated that the authors performed many important control experiments such as the FACS analysis of IFNAR expression in IFNAR1deltaNKp46, IFNAR1deltaVav1 and IFNAR1fl/fl mice. The manuscript is very well written.

Reviewer #3: The viral pathogen ectromelia virus ECTV is causing mousepox, a disease which is of variable clinical outcome depending on the strain of mice used. C57BL/6 mice are naturally resistant and survive without disease, while broadly IFNAR-deficient B6 mice rapidly die. In this manuscript by Melo-Silva et al., the authors expand on this finding and explore the effect of tissue-specific IFNAR-deficiency on survival in the ECTV foodpad infection model. They elegantly show that IFNAR expression on NK cells and monocytes, more specifically iMOs, is required for protection, while IFNAR signalling in T cells seems dispensible. The finding that NK cells were less cytolytic and iMOs less efficient in restricting viral dissemination due to IFNAR deficiency in this model is concincingly demonstrated. The expendable role of intrinsic IFNAR signalling for T cell generation/activation is less clear. The manuscript is very well written and the experiments are thoroughly performed. The manuscript contains interesting and novel data. I have however some points that should be addressed.

**Part II – Major Issues: Key Experiments Required for Acceptance**

Please use this section to detail the key new experiments or modifications of existing experiments that should be absolutely required to validate study conclusions.

Generally, there should be no more than 3 such required experiments or major modifications for a "Major Revision" recommendation. If more than 3 experiments are necessary to validate the study conclusions, then you are encouraged to recommend "Reject".

**Reviewer #1:**

(1) Figure 3G-3K indicate that the absence of IFNAR in the T cells is not detrimental for protective responses using the proportions of reconstituted bone marrow cells indicated (20%). What is the rationale for this proportion? Have these proportions been titrated? Could there be a mixture (let’s say 90% RAG + 10% IFNAR or B6) at which the absence of IFNAR makes the T cell response worse?

To make the mixed bone marrow chimeras, we used the exact same reconstitution protocol previously used by Michael Diamond’s lab [1]. In the referenced paper, the authors titrated different ratios of *Rag2*^-/-^: *Ifnar1*^-/-^ cells for reconstitution and their conclusion was that 80% *Rag2*^-/-^: 20% *Ifnar1*^-/-^ cells was the ideal ratio to fully reconstitute the T cell compartment of *Rag1*^-/-^ + *Ifnar1*^-/-^→ *Rag1*^-/-^ mice with *Ifnar1^-/-^* T cells, while maintaining a majority of Ifnar^+/+^ myeloid cells. They observed that reconstituting irradiated mice with a 90% *Rag2*^-/-^: 10% *Ifnar1*^-/-^ cells did not consistently result in normal numbers of T cells, suggesting that with this ratio, the frequency of *Ifnar1*^-/-^ stem cells in the bone marrow transplant may not suffice to properly reconstitute the T cell compartment. Therefore, we decided to adopt the 80%:20% ratio in our experiments. As reported by the Diamond lab, we found that the T cell compartment was well reconstituted in these mice. Given Diamond’s publication, we think it is unnecessary to show these results. We now provide the reference to the Diamond paper when we describe the model.

(2) It would be informative to measure the actual amounts of IFN in the dLN of IFNAR-delta-Lyz2 animals compared to IFNAR-fl/fl animals. If IFNAR is needed in iMO to enhance IFN-I levels rather than provide antiviral signalling, the defect of IFNAR-delta-Lyz2 animals should be by-passed by exogenous administration of physiological levels of IFN (i.e., resembling those observed in IFNAR-fl/fl animals).

In response to the first comment, we made various attempts to measure actual levels of IFN-I in the dLN of *Ifnar1*^ΔLyz2^ mice. For this, we used a reporter cell line (ISRE-Luc L9 cells) as we did in a previous paper [2]. Unfortunatelly, after these many years, the ISRE-Luc L9 cells in our LN2 tank seem to have lost the ability to produce Luciferase in response to IFN-I. For these experiments, we used all our stock of *Ifnar1*^ΔLyz2^ mice. Thus, we currently do not have additional mice to try other methods in a timely manner. In addition, our funding for this project has ended and we no longer have the resources to expand the colony or attempt expensive methods such as Luminex. Nevertheless, we think that the proposed experiment will not necessarily be informative becuase we expect that *Ifnar1*^ΔLyz2^ mice will produce IFN-I in the dLN. Our previous published data (Figure 7 in reference [3]) showed that *Ifnar1^-/-^* mice transcribed IFN-I in the dLN in response to ECTV. Indeed, the dLNs of *Ifnar1^-/-^* mice transcribed significantly more IFN-I than the dLN of WT mice. However, virus loads (as determined by viral transcripts) were ~50-fold higher in the dLN of *Ifnar1^-/-^* than WT mice. These results suggest that high virus loads can bypass the requirement for positive IFN-I feedback. Figure 6D of the current paper shows that viral loads are increased 10-100 fold in the dLN of *Ifnar1*^ΔLyz2^ mice at 3dpi (Figure 6D). This high virus load introduces a new variable that would likely result in high IFN-I transcription as in plain *Ifnar1^-/-^* mice. It was exactly to avoid this specific problem why we used the bone marrow chimera approach in Figure 7 of this paper. In these chimeras, WT and *Ifnar1^-/-^* iMOs were obtained from the same dLN, and were therefore exposed to the same virus load. Thus, the only variable was intrinsic IFNAR1 expression. This revealed the existence of the positive feedback loop. Of note, Figure 7B shows that IFN-I feedback in iMOs is required for optimal IFN-I production. However, this requirement is not absolute because *Ifnar1^-/-^* iMOs did upregulate IFN-I compared to naïve MOs. We have modified Figure 7B and the text to make this point more clear.

In response to the suggestion to add back IFN-I, we think this type of experiment is interesting and worth exploring, but is not essential to the message of this paper. These experiments would require extensive time and resources because it would be necessary to empirically determine the IFN-I subtype, dose and route to rescue the mice. We hope we can perform these experiments in the future, once we recover our funding.

**Reviewer #2:** (No Response)

**Reviewer #3:**

The authors discuss and expand the relevance of their findings to other viral pathogens and infection models. This however has strong limitations since the footpad infection model is a highly artificial one and does not represent the natural infection route for ECTV. I think the data would be more relevant if the authors could reproduce at least some of their findings in a clinically relevant route for mousepox infection e.g. the intranasal route for respiratory infection. This would exclude route-specific effects and, additionally, support the role of this model as a surrogate model for human smallpox.

We agree that it would be interesting to test additional infection routes. However, as it stands, the paper already has seven multi-panel figures and two supplementary figures. We kindly disagree with the reviewer regarding the assertion that the footpad infection model is artificial and does not represent the natural route of ECTV infection. Whereas intranasal infection is thought to be the natural route of infection for variola virus and possibly monkeypox virus in humans, extensive work has shown that ECTV naturally enters its mouse host through microabrasions in the skin of the footpad and then spreads lymphhematogenously [4]. In fact, ECTV *via* the footpad is one of the textbook examples for lymphohematogenous virus dissemination within the host [5]. We acknowledge the value of using intranasal challenge with ECTV to mimic human smallpox and other respiratory infections. However, our laboratory uses ECTV’s natural route as an acute lethal challenge model for viruses that dissiminate lymphohematogenously.

**Part III – Minor Issues: Editorial and Data Presentation Modifications**
Please use this section for editorial suggestions as well as relatively minor modifications of existing data that would enhance clarity.

**Reviewer #1:**

(3) It would be good to emphasise in the discussion that the dispensable role of IFNAR in T cell responses is observed in the context of primary infection and that, although the authors measure CD44+ cells at 22 dpi, results could be different in the context of challenge or others.

As suggested by the reviewer, we discuss in the manuscript that our conclusions are restricted to the acute phase of infection. It should be noted, however, that others have shown that *Ifnar1^-/-^* mice can be protected from lethal mousepox by vaccination with attenuated ECTV [6]. We now make reference to this paper in the discussion.

(4) A previous paper from the group in PLOS Pathogens showed that cGAS deficiency in parenchymal cells did not affect IFN-I production and resistance to mousepox (being cGAS the main IFN-producing sensor for ECTV). This is now extended here showing that IFNAR is also dispensable in parenchymal cells. Could this poor immune function of hepatocytes explain why the liver is a preferred target organ for ECTV?

The reviewer comment is intriguing. Even though hepatocytes do not seem to play a primordial role in IFN-I production based on our preceding published work [3], we have also previously demonstrated the presence of IFN-I in susceptible infected animals in the liver [2]. It is unclear whether the IFN-I found in the liver is produced by parenchymal or by resident or infiltrating immune cells. Therefore, we do not know whether a poor immune function of hepatocytes causes the liver to be a preferred target organ. We now speculate about this possibility in the discussion.

(5) In this same previous paper, IFN-I expression in the dLN was unaffected by the absence of IFNAR, which seemingly contradicts the findings presented here. What is the authors’ explanation for these observations?

This point is explained in more detail in the reply to Reviewer #1, point 2, in the Major Issues Section. Briefly, *c*ompared to the mixed bone marrow chimeras, *Ifnar1^-/-^* mice experience very high virus loads which we belive bypasses the need for intrinsic IFNAR signaling for optimal IFN-I transcription.

(6) The IFN-I/IFNAR positive loop is somewhat surprising because IFN itself is not an ISG. However, one could think of indirect mechanisms by which other ISG contribute to enhanced IFN production, like IRF7 or cGAS. The authors should elaborate on this in the Discussion.

We agree with this comment. Indeed, in experiments for a paper soon to be submitted, we observed that *Ifnar1*^-/-^ sorted iMOs from dLNs of bone marrow chimeras have reduced transcription of *cGas*, *Irf7* and other ISGs as determined by mRNA Seq (of note, this mRNA seq also confirmed the results in Figure 7 for IFN-I genes). As suggested by the reviewer, we now discuss these possibilities in the text.

(7) The paper is well-written and the data are well-presented, with some minor formatting issues that the production team should easily sort out.

**Reviewer #2:** (No Response)

**Reviewer #3:**

Could the authors speculate more about the role of IFN I on T cell responses in the ECTV infection model? I think there are known inhibitors of IFN I in ECTV most likely interfering with T cell generation anyway and this must be compensated somehow (e.g. by IL-12 as has been shown for vaccinia VACV?).

We have previously shown that the ECTV IFN-α decoy receptor EVM166 is essential for ECTV virulence in WT B6 mice but not in *Ifnar1^-/-^* mice [2, 7]. Thus, there is a delicate equilibrium between IFN-I antagonism by the virus and IFN-I production by the host. Other cytokines also contribute to resistance as IL12/IL-18 double KO, but not single mutant mice are susceptible to lethal mousepox [8]. We now discuss other cytokines as possible alternative sources of Signal 3.

In this context, the authors should also restrict the relevance of their data to the acute phase of infection. Memory responses have not been investigated here, although IFN I has a known strong and particular effect on memory cell formation in various other viral infection models.

Please see reply to Reviewer #1 (3) in Minor Comments.

In this study, T cell responses have been exclusively investigated systemically in spleen and blood. Since the foodpad route is a strictly peripheral one, the T cell responses in the dLNs would be of particular interest, especially with regard to Figure 6 H-J.

We did not focus our analysis of the T cell response in the dLN because it is still absent at 1-3 dpi. The dLN becomes highly necrotic at 4-5 dpi during primary infection and the analysis of T cell responses is therefore not possible at these times. T cell responses can be detected in the dLN at 4-5 but not 1-3 dpi only in mice that have been previously immunized.

Fig. 4H GzmB on x axis is not ledgible.

Thanks. We have corrected this mistake.

**References**

1.	Diamond MS, Kinder M, Matsushita H, Mashayekhi M, Dunn GP, Archambault JM, et al. Type I interferon is selectively required by dendritic cells for immune rejection of tumors. J Exp Med. 2011;208(10):1989-2003. Epub 2011/09/21. doi: 10.1084/jem.20101158. PubMed PMID: 21930769; PubMed Central PMCID: PMCPMC3182061.

2.	Xu RH, Rubio D, Roscoe F, Krouse TE, Truckenmiller ME, Norbury CC, et al. Antibody inhibition of a viral type 1 interferon decoy receptor cures a viral disease by restoring interferon signaling in the liver. PLoS Pathog. 2012;8(1):e1002475. Epub 2012/01/14. doi: 10.1371/journal.ppat.1002475. PubMed PMID: 22241999; PubMed Central PMCID: PMCPMC3252373.

3.	Wong EB, Montoya B, Ferez M, Stotesbury C, Sigal LJ. Resistance to ectromelia virus infection requires cGAS in bone marrow-derived cells which can be bypassed with cGAMP therapy. PLoS Pathog. 2019;15(12):e1008239. Epub 2019/12/27. doi: 10.1371/journal.ppat.1008239. PubMed PMID: 31877196; PubMed Central PMCID: PMCPMC6974301.

4.	Fenner F. Mouse-pox; infectious ectromelia of mice; a review. J Immunol. 1949;63(4):341-73. Epub 1949/12/01. PubMed PMID: 15398116.

5.	Sigal LJ. The Pathogenesis and Immunobiology of Mousepox. Adv Immunol. 2016;129:251-76. Epub 2016/01/23. doi: 10.1016/bs.ai.2015.10.001. PubMed PMID: 26791861.

6.	Panchanathan V, Chaudhri G, Karupiah G. Interferon function is not required for recovery from a secondary poxvirus infection. Proc Natl Acad Sci U S A. 2005;102(36):12921-6. Epub 2005/08/27. doi: 10.1073/pnas.0505180102. PubMed PMID: 16123129; PubMed Central PMCID: PMCPMC1200282.

7.	Xu RH, Cohen M, Tang Y, Lazear E, Whitbeck JC, Eisenberg RJ, et al. The orthopoxvirus type I IFN binding protein is essential for virulence and an effective target for vaccination. J Exp Med. 2008;205(4):981-92. Epub 2008/04/09. doi: 10.1084/jem.20071854. PubMed PMID: 18391063; PubMed Central PMCID: PMCPMC2292233.

8.	Wang Y, Chaudhri G, Jackson RJ, Karupiah G. IL-12p40 and IL-18 play pivotal roles in orchestrating the cell-mediated immune response to a poxvirus infection. J Immunol. 2009;183(5):3324-31. Epub 2009/08/07. doi: 10.4049/jimmunol.0803985. PubMed PMID: 19657092.
